# Characterization of NiCas12b for In Vivo Genome Editing

**DOI:** 10.1002/advs.202400469

**Published:** 2024-07-30

**Authors:** Yunqian Zhang, Jingjing Wei, Hongyan Wang, Yongming Wang

**Affiliations:** ^1^ Shanghai Key Laboratory of Metabolic Remodeling and Health Institute of Metabolism and Integrative Biology State Key Laboratory of Genetic Engineering at School of Life Sciences Fudan University Shanghai 200438 China; ^2^ Center for Medical Research and Innovation Shanghai Pudong Hospital Fudan University Pudong Medical Center Fudan University Shanghai 200438 China; ^3^ Obstetrics & Gynecology Hospital State Key Laboratory of Genetic Engineering Shanghai Key Laboratory of Metabolic Remodeling and Health Institute of Metabolism and Integrative Biology Fudan University Shanghai 200438 China; ^4^ Children's Hospital Fudan University Shanghai 201102 China; ^5^ Shanghai Engineering Research Center of Industrial Microorganisms Shanghai 200438 China

**Keywords:** CRISPR‐Cas, genome editing, NiCas12b, PAM, PCSK9

## Abstract

The RNA‐guided clustered regularly interspaced short palindromic repeats (CRISPR)/Cas12b system represents the third family of CRISPR‐Cas systems that are harnessed for genome editing. However, only a few nucleases have demonstrated activity in human cells, and their in vivo therapeutic potential remains uncertain. In this study, a green fluorescent protein (GFP)‐activation assay is conducted to screen a panel of 15 Cas12b orthologs, and four of them exhibited editing activity in mammalian cells. Particularly noteworthy is the NiCas12b derived from *Nitrospira sp*., which recognizes a “TTN” protospacer adjacent motif (PAM) and facilitates efficient genome editing in various cell lines. Importantly, NiCas12b also exhibits a high degree of specificity, rendering it suitable for therapeutic applications. As proof of concept, the adeno‐associated virus (AAV) is employed to introduce NiCas12b to target the cholesterol regulatory gene proprotein convertase subtilisin/ kexin type 9 (*Pcsk9*) in the mouse liver. After 4 weeks of injections, an impressive is observed over 16.0% insertion/deletion (indel) efficiency, resulting in a significant reduction in serum cholesterol levels. NiCas12b provides a novel option for both basic research and clinical applications.

## Graphical abstract

Using a GFP‐activation assay to screen a panel of Cas12b orthologs, four are identified that exhibit editing activity in mammalian cells. Among them, NiCas12b, derived from *Nitrospira sp*., recognizes a TTN protospacer adjacent motif (PAM) and facilitates efficient genome editing in both cell lines and mice. NiCas12b also demonstrates a high degree of specificity, making it suitable for therapeutic applications.

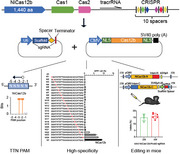

## 1 Introduction

The clustered regularly interspaced short palindromic repeats‐Cas (CRISPR‐Cas) system, originating from microbial adaptive immune systems, has been widely harnessed for genome editing in both basic research and gene therapy.^[^ ^1^, ^2^, ^3^ ^]^ This system is classified into two classes, comprising 6 types and over 30 subtypes, based on functional and evolutionary modularity.^[^ ^4^ ^]^ Among these, Class 2, including type II (encoding Cas9) and type V (encoding Cas12), CRISPR‐Cas systems have been predominantly used for genome editing due to their reliance on a single effector protein for DNA cleavage.^[^ ^5^, ^6^, ^7^, ^8^, ^9^ ^]^

The first Cas9 system, SpCas9, was developed for mammalian genome editing in 2013 and has since become the most popular genome editing tool.^[^ ^1^ ^]^ SpCas9 has been successfully applied in a wide range of organisms, including bacteria,^[^ ^10^ ^]^ yeast,^[^ ^11^ ^]^ plants,^[^ ^12^ ^]^ mice,^[^ ^13^ ^]^ and humans.^[^ ^14^ ^]^ Type V‐A Cas12a is the second CRISPR‐Cas system that was developed in 2015.^[^ ^5^ ^]^ Subsequently, numerous CRISPR‐Cas systems from various bacteria have been engineered for genome editing.^[^ ^15^, ^16^, ^17^, ^18^ ^]^ These CRISPR‐Cas nucleases exhibit distinct properties, such as protospacer adjacent motif (PAM) requirements,^[^ ^9^, ^18^ ^]^ target sequence preferences,^[^ ^8^, ^19^ ^]^ and specificity.^[^ ^7^, ^20^ ^]^ However, it is important to note that there are still many genomic loci that cannot be efficiently edited due to the lack of an appropriate PAM or because the sequence is not preferred by the current CRISPR‐Cas tools. Hence, there is a pressing need to expand the CRISPR‐Cas toolbox to broaden the scope of genome editing possibilities.

Type V‐B Cas12b represents the third family of CRISPR‐Cas systems that have been utilized for genome editing. This system consists of three key components: a Cas12b nuclease, a CRISPR RNA (crRNA), and a trans‐activating crRNA (tracrRNA).^[^ ^21^ ^]^ The crRNA and tracrRNA can be fused together to create a single guide RNA (sgRNA).^[^ ^21^ ^]^ Up to now, three Cas12b nucleases (AaCas12b,^[^ ^22^ ^]^ BhCas12b v4,^[^ ^23^ ^]^ and BvCas12b^[^ ^23^ ^]^) have demonstrated activity in mammalian cells, although their in vivo therapeutic potential has not been investigated. In this study, we conducted a screening of a total of 15 Cas12b orthologs and identified NiCas12b as an efficient and highly specific enzyme for genome editing. Importantly, our research demonstrates that NiCas12b can disrupt the mouse proprotein convertase subtilisin/ kexin type 9 (*Pcsk9*) gene in vivo, showcasing significant promise for therapeutic applications.

## 2 Results

### 2.1 Selection of Cas12b Orthologs

We utilized AaCas12b as a reference to identify related orthologs from the NCBI database. We selected 15 orthologs with complete Cas12b coding genes, direct repeats, and tracrRNA (**Table** **1**). The length of Cas12b in these orthologs ranged from 1064 aa to 1529 aa, and their identity to AaCas12b varied from 23.1% to 65.5%. Phylogenetic analysis revealed a close relationship among four newly identified Cas12b orthologs (AhCas12b, AlicCas12b, ThCas12b, and PpCas12b) and previously characterized three Cas12b orthologs (BvCas12b, BhCas12b, and AaCas12b), as they clustered together (Figure S1, Supporting Information). The remaining 11 Cas12b orthologs were clustered distinctively, away from the aforementioned three previously characterized Cas12b orthologs (Figure S1, Supporting Information). These findings underscore the diverse nature of the Cas12b system in the natural environment.

**Table 1:** Fifteen Cas12b orthologs were selected from the NCBI database.

Name	NCBI accession	Host strain	Subject length	Identity to AaCas12b [%]
AhCas12b	MCL6632093.1	Alicyclobacillus herbarius	1149	65.5
AlicCas12b	MCL6517083.1	Alicyclobacillus sp.	1092	61.3
AlpCas12b	MBM3539290.1	Alphaproteobacteria bacterium	1120	36.9
BryCas12b	MCL4782191.1	Bryobacterales bacterium	1471	23.1
DtCas12b	WP_163 299 037.1	Dissulfurirhabdus thermomarina	1529	23.6
GeoCas12b	MBI1922408.1	Geobacter sp.	1096	34.8
LacCas12b	MBQ5561364.1	Lachnospiraceae bacterium	1064	31.2
LepCas12b	MCB1144584.1	Leptospiraceae bacterium	1333	28.4
NiCas12b	MCP9456315.1	Nitrospira sp.	1440	25.4
PhoCas12b	WP_255 045 201.1	Photobacterium sp. ZSDE20	1182	29
PhyCas12b	QOJ17852.1	Phycisphaera sp. HKU‐PLA1	1459	24.8
PpCas12b	WP_188 494 874.1	Pullulanibacillus pueri DSM 100927	1131	41.2
SynCas12b	MBP9020642.1	Syntrophobacterales bacterium	1353	23.1
ThCas12b	MBX6353558.1	Thermoflavifilum sp.	1127	62.5
TurCas12b	MBX3721360.1	Turneriella sp.	1276	31.1

Next, we conducted an analysis of the genomic architecture of CRISPR‐Cas12b. Among the orthologs, 11 shared a consistent arrangement, featuring Cas12b followed by Cas1, Cas2, tracrRNA, and a CRISPR array (Figure S2, Supporting Information). The number of spacers within this set ranged from 1 to 106 (Figure S2, Supporting Information). Alignment of repeat and tracrRNA sequences indicated a moderate level of conservation (Figure S3a,b, Supporting Information). Notably, 13 repeats displayed a conserved CAC sequence at the 3′ end. We fused the 3′ end of the tracrRNA sequence to the 5′ end of the repeat sequence to form a sgRNA. In silico analysis using UNAFold^[^ ^24^ ^]^ revealed that the majority of sgRNAs formed a similar secondary structure, characterized by three stem loops, typically with a bulge at the second stem loop (Figure S4, Supporting Information). This bulge often contained nucleotides capable of pairing with the 3′ end of the repeat sequence. Collectively, these findings underscore the conservation of Cas12b guide RNA.

### 2.2 Test of Cas12b Ortholog Activity

**Figure 1:**
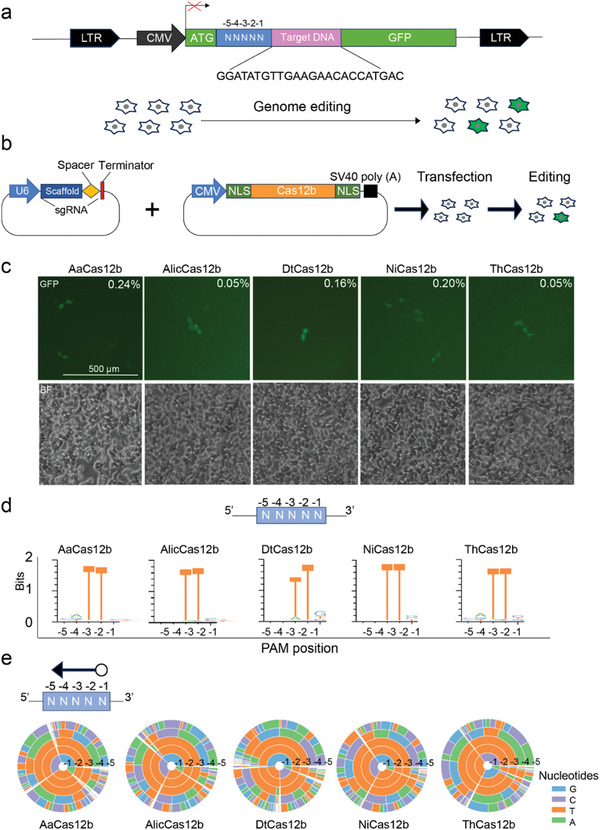
Test of Cas12b activity for mammalian genome editing. a) Schematic of the GFP‐activation assay designed to assess the activity of Cas12b orthologs. A target DNA sequence with a 5‐bp random sequence at its 5′ end is inserted between the translation initiation codon (ATG) and the remaining part of the GFP coding sequence, resulting in a frame‐shift mutation. The reporter library is stably integrated into human HEK293T cells. Genome editing that induces indels at the target sequence results in GFP expression due to the re‐establishment of the open reading frame in a subset of cells. CMV, cytomegalovirus. LTR, long terminal repeat. b) The Cas12b expression plasmid and the sgRNA expression plasmid are co‐transfected into reporter cells for genome editing. c) Transfection of Cas12b with sgRNA leads to GFP expression, with AaCas12b being used as a positive control. BF, bright field. d) WebLogo diagrams are generated based on deep sequencing data for AaCas12b, AlicCas12b, DtCas12b, NiCas12b, and ThCas12b. e) PAM wheels are generated based on deep sequencing data for AaCas12b, AlicCas12b, DtCas12b, NiCas12b, and ThCas12b.

To evaluate the activities of the Cas12b orthologs, we employed a green fluorescent protein (GFP)‐activation assay.^[^ ^6^ ^]^ In this assay, a 5‐bp random sequence followed by a 24‐bp target sequence (protospacer) was inserted between the translation initiation codon (ATG) and the GFP coding sequence, inducing a frameshift mutation (**Figure** **1**a). This construct was stably integrated into HEK293T cells using lentivirus. If a Cas12b ortholog successfully edits the target, it leads to the generation of insertions or deletions (indels), causing in frame mutations in a subset of cells and subsequent GFP expression. For each Cas12b ortholog, we optimized it for human codon usage, synthesized it, and cloned it into a mammalian expression construct. The sgRNA was expressed on a separate plasmid (Figure 1b). AaCas12b was used as a positive control. Five days after transfecting the Cas12b and sgRNA expressing plasmids into the reporter cells, GFP expression was observed for five of the orthologs: AaCas12b, AlicCas12b, DtCas12b, NiCas12b, and ThCas12b (Figure 1c).

The GFP‐positive cells were separated using flow cytometry, and the target sequence, which included the 5‐bp random sequence, was amplified via polymerase chain reaction (PCR) for deep sequencing analysis. Further analysis using the WebLogo^[^ ^25^ ^]^ diagram and the PAM wheel^[^ ^26^ ^]^ revealed that AaCas12b recognized a TTN PAM (Figure 1d,e), consistent with a previous study.^[^ ^22^ ^]^ Additionally, the four new orthologs also displayed a preference for recognizing a TTN PAM, suggesting that the Cas12b family recognizes a conserved PAM sequence.

### 2.3 Optimization of sgRNA

Next, we conducted tests to determine the optimal spacer (guide) length for each Cas12b ortholog targeting the AAVS1 locus (Figure S5a, Supporting Information). Spacers ranging from 19 to 26 nucleotides were designed, revealing that 20–21 nucleotide spacers exhibited higher activity for AlicCas12b and ThCas12b, while 21–26 nucleotide spacers displayed higher activity for DtCas12b and NiCas12b (Figure S5b, Supporting Information).

To enhance editing activities, we engineered sgRNA scaffolds by truncating stem loops or removing bulges and evaluated their activity targeting the AAVS1 locus. Specifically, for AlicCas12b, truncating stem‐loop 3 resulted in reduced editing efficiency (Figure S6a,b, Supporting Information). In the case of DtCas12b, removing the bulge on stem‐loop 3 (V1) or truncating stem‐loop 2 (V2) led to decreased editing efficiency (Figure S7a,b, Supporting Information). Only truncation of stem‐loop 3 (V3) had no impact on editing efficiency and was subsequently used in subsequent studies (Figure S7a,b, Supporting Information).

Modifications for NiCas12b sgRNA scaffold included: V1–removing the bulge on stem‐loop 2; V2—removing the bulge on stem‐loop 2 and truncating stem‐loop 2; V3—truncating stem‐loop 3; V4—removing the bulge on stem‐loop 2 and truncating stem‐loop 3 (Figure S8a, Supporting Information). V1, V2, and V4 modifications dramatically reduced editing efficiency, while V3 modification increased efficiency and was chosen for subsequent studies (Figure S8b, Supporting Information). For ThCas12b, truncating stem‐loop 3 resulted in reduced editing efficiency (Figure S9a,b, Supporting Information). The above results emphasized the crucial role of stem‐loop 2, which should remain unchanged. In contrast, truncating stem‐loop 3 at an appropriate position may preserve editing efficiency.

### 2.4 Test of Three Cas12b Ortholog Activity

**Figure 2:**
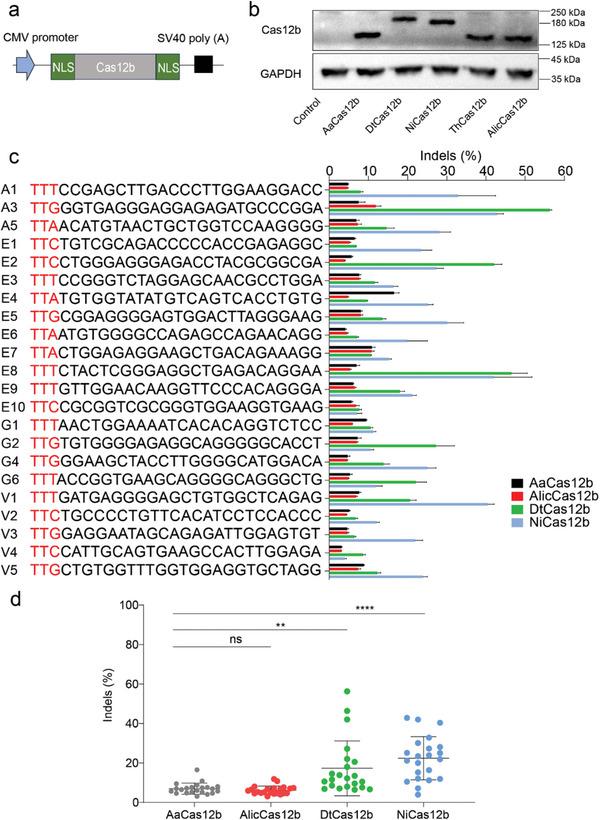
Analysis of Cas12b activity. a) Schematic diagram of the Cas12b expression constructs. All five Cas12b expression constructs share the same backbone. Poly (A), polyadenylated. b) Expression levels of AaCas12b, AlicCas12b, DtCas12b, ThCas12b, and NiCas12b are analyzed by western blot. The glyceraldehyde‐3‐phosphate dehydrogenase (GAPDH) is used for a positive control. c) The editing efficiency of AaCas12b, AlicCas12b, DtCas12b, and NiCas12b in HEK293T is measured by targeted deep sequencing (*n* = 3). The sequences of target sites are shown on the left, with PAMs marked in red. AaCas12b, DtCas12b, and NiCas12b use 24‐nt spacers, while AlicCas12b uses 21‐nt spacers. d) Quantification of editing efficiencies in HEK293T cells. For posthoc analysis, a repeated‐measures (RM) one‐way analysis of variance (ANOVA) test followed by Fisher's least significant difference test is used. A value of *p* < 0.05 was considered to be statistically significant (*p < 0.05, **p < 0.01, ***p <0.001, and ****p < 0.0001).

Next, we tested the activity of AlicCas12b, DtCas12b, and NiCas12b, with AaCas12b being used as a control. All five Cas12b orthologs were expressed from the same construct backbone (**Figure** **2**a). Western blot analysis demonstrated similar gene expression levels for all four Cas12b orthologs (Figure 2b). We excluded ThCas12b because it displayed low activity at the AAVS1 locus (Figure S5b, Supporting Information).

We selected a panel of 22 endogenous loci containing TTN PAMs and tested the activities of these Cas12b orthologs in HEK293T cells. All four Cas12b nucleases induced indels over 3.1% at the selected sites (Figure 2c). Overall, the average activities of these orthologs ranked as follows: NiCas12b > DtCas12b > AaCas12b > AlicCas12b (Figure 2d). Interestingly, these orthologs exhibited distinct sequence preferences. For instance, NiCas12b displayed higher activity than DtCas12b at the sites A1, A5, E5, and V1, while DtCas12b exhibited higher activity than NiCas12b at the sites A3, E2, and G2. These data underscore the importance of expanding the CRISPR‐Cas toolbox for efficient genome editing.

We further tested the activity of these orthologs with a panel of nine loci in mouse Neuro‐2a (N2a) cells and observed a similar rank of activity (Figure S10a,b, Supporting Information). Given that NiCas12b demonstrated higher activity than other orthologs, we focused on NiCas12b in the subsequent study. To further evaluate NiCas12b activity, we tested NiCas12b activity at six endogenous loci in additional cell types, including HeLa, HCT116, and AC16. NiCas12b induced indels in all these cell types, with efficiencies ranging from 22.6% to 84.4% (Figure S11a–c, Supporting Information). This demonstrates that NiCas12b functions with high efficiency across a variety of cell types.

### 2.5 Analysis of NiCas12b Specificity

**Figure 3:**
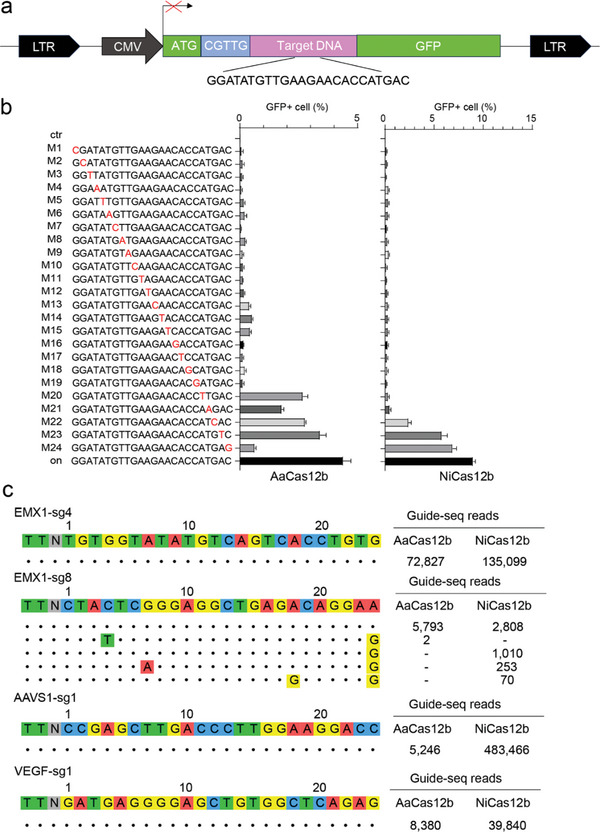
Analysis of NiCas12b specificity. a) The schematic diagram illustrates the GFP‐activation assay utilized for specificity analysis. b) On the left, a panel of sgRNAs with single‐nucleotide mutations is presented, with mismatches being highlighted in red. The activity of each sgRNA for NiCas12b and AaCas12b is analyzed based on GFP expression (*n* = 3). c) For four loci, off‐targets are analyzed by GUIDE‐seq. Read numbers for both on‐ and off‐targets are provided on the right, with mismatches being compared with the on‐target site indicated in color.

Next, we assessed the specificity of NiCas12b utilizing the GFP‐activation assay (**Figure** **3**a), with AaCas12b being employed as a control. To investigate off‐target effects, a set of 24 spacers with single nucleotide mismatches was generated alongside the design of an on‐target sgRNA for comparison. The cleavage efficiency, reflected by the percentage of GFP‐positive cells, was analyzed 3 days post‐transfection of Cas12b and sgRNA‐expressing plasmids into reporter cells using fluorescence activated cell sorting (FACS). The results demonstrated that both AaCas12b and NiCas12b exhibited high specificity. AaCas12b generated minimal levels of GFP‐positive cells when a single mismatch occurred at PAM‐proximal positions 1–19 (Figure 3b). Similarly, NiCas12b displayed minimal levels of GFP‐positive cells when a single mismatch occurred at PAM‐proximal positions 1–21 (Figure 3b). These data underscore the remarkable sensitivity of Cas12b nucleases to single mismatches.

Additionally, we conducted a genome‐wide analysis of off‐target effects for NiCas12b using GUIDE‐seq,^[^ ^27^ ^]^ with AaCas12b serving as a comparative reference. Two sgRNAs targeting the EMX1 gene, one targeting the AAVS1 locus, and one targeting the VEGFA gene were designed. Five days post co‐transfection of the Cas12b/sgRNA‐expressing plasmid with GUIDE‐seq oligos into HEK293T cells, we collected the cells and prepared libraries for deep sequencing. The sequencing results revealed efficient on‐target cleavage for both AaCas12b and NiCas12b, evidenced by the high GUIDE‐seq read counts (Figure 3c). AaCas12b generated one off‐target for the EMX1 locus (sg8), while NiCas12b produced three off‐targets for the EMX1 locus (sg8). No off‐target effects were detected for the other three loci. These findings affirm that both AaCas12b and NiCas12b nucleases exhibit high specificity in genome editing.

### 2.6 Allele‐Specific Genome Editing with NiCas12b

Several studies have demonstrated that autosomal dominant diseases can be treated by nonhomologous end‐joining (NHEJ) gene disruption of mutant alleles.^[^ ^28^, ^29^ ^]^ To test the capability of NiCas12b for allele‐specific genome editing, we selected six dominant‐negative disease‐causing mutations. These included two mutations from F11, which induce Factor XI (FXI) deficiency;^[^ ^30^ ^]^ two mutations from TP53, which induce human cancer;^[^ ^31^ ^]^ one mutation from KCNC3, which induces familial ataxia;^[^ ^32^ ^]^ and one mutation from MYH7, which induces hypertrophic cardiomyopathy.^[^ ^33^, ^34^ ^]^ SpCas9 was used for comparison. We synthesized a DNA fragment containing these mutations and inserted it into the chromosome using lentivirus in HEK293T cells (Figure S12a,b, Supporting Information). We then designed sgRNAs for both SpCas9 and NiCas12b to target these mutations.

Seven days after genome editing, we tested the rates of indels at both the integrated sites (mutant allele) and the endogenous wild‐type (WT) sites. The results showed that SpCas9 and NiCas12b induced indels at the target sites (Figure S13, Supporting Information). Additionally, SpCas9 induced substantial indels at the endogenous sites, while NiCas12b induced negligible indels at these sites. Notably, NiCas12b displayed higher efficiency at three loci (*MYH7* c. 767G>A, *TP53* c. 733G>A, and *TP53* c. 742C>T). These data demonstrate the advantages of NiCas12b over SpCas9 for allele‐specific genome editing.

### 2.7 Design of Intein‐Mediated Split‐NiCas12b to Target Mouse *Pcsk9*

**Figure 4:**
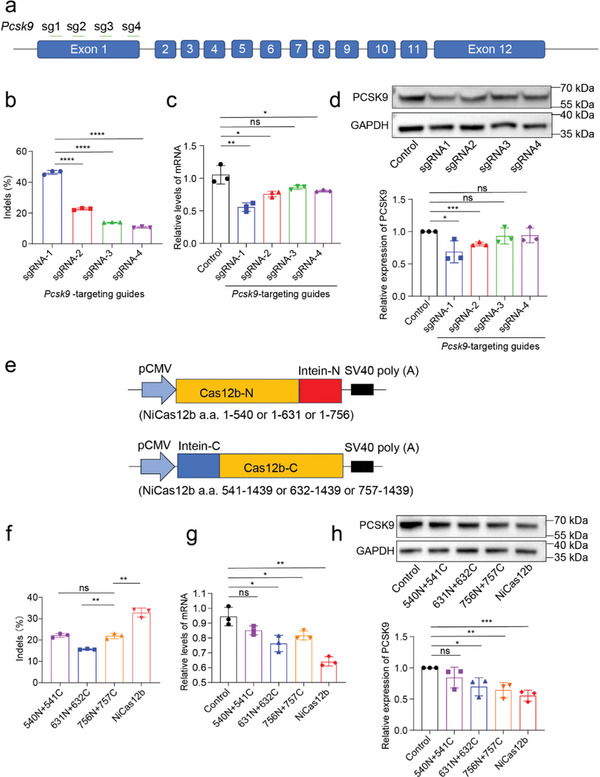
Evaluation of intein‐mediated split‐NiCas12b in mouse Hepa1‐6 cells. a) Schematic diagram of the mouse *Pcsk9* gene. The sgRNA target loci are shown on the top. sg1, sgRNA‐1; Sg2, sgRNA‐2; sg3, sgRNA‐3; sg4, sgRNA‐4. b) After 5 days of editing using the full‐length NiCas12b, the indel efficiencies are determined in Hepa1‐6 cells. c) Expression levels of *Pcsk9* mRNA relative to GAPDH are measured by reverse transcription quantitative PCR (RT‐qPCR) after genome editing. d) Expression levels of PCSK9 are measured by western blot. The GAPDH is used as a positive control. e) The intein‐mediated split‐NiCas12b is expressed in two plasmids. Here, the split sites are shown. The split sites are chosen between 540Ser and 541Asn (540N+541C), 631Lys and 632Ala (631N+632C), or 756Lys and 757Thr (756N+757C). f) After 5 days of editing using intein‐mediated split‐NiCas12b and sgRNA‐1, the indel efficiencies are determined in Hepa1‐6 cells. The full‐length NiCas12b is used for a positive control. g) Expression levels of *Pcsk9* mRNA relative to GAPDH are measured by reverse transcription quantitative PCR (RT‐qPCR) after editing with three intein‐mediated split‐NiCas12b. h) Expression levels of PCSK9 are measured by western blot with three intein‐mediated split‐NiCas12b. The GAPDH is used as a positive control. A value of *p* < 0.05 was considered to be statistically significant (*p < 0.05, **p < 0.01, ***p <0.001, and ****p < 0.0001).

Cas12b has not been utilized for in vivo genome editing. To assess the therapeutic potential of NiCas12b, we utilized it to disrupt *Pcsk9* in the mouse liver. PCSK9 acts as a negative regulator of the low‐density lipoprotein (LDL) receptor and is primarily expressed in the liver.^[^ ^35^ ^]^ Inhibiting PCSK9 has proven effective in reducing serum cholesterol levels and holds promise for reducing the risk of coronary heart disease.^[^ ^2^ ^]^ We designed four sgRNAs targeting *Pcsk9* exon 1 and tested their activity in murine hepatoma cells (Hepa1‐6, **Figure** **4**a). Five days after transfection of NiCas12b and sgRNA expressing plasmids into Hepa1‐6 cells, followed by puromycin selection, genomic DNA was isolated for targeted deep sequencing. The sequencing results demonstrated that all sgRNAs induced indels at the target sites, with sgRNA1 being the most efficient (Figure 4b). Reverse transcription quantitative PCR (RT‐qPCR) revealed a decrease in *Pcsk9* messenger RNA (mRNA) levels following genome editing (Figure 4c). Western blot analysis confirmed a reduction in PCSK9 protein levels after genome editing (Figure 4d). Based on these results, sgRNA1 was selected for subsequent experiments.

NiCas12b, due to its large genome exceeding the ≈4.7 kb packing capacity of adeno‐associated virus (AAV), presented a challenge for in vivo genome editing. To overcome this limitation, we employed intein technology for splitting, inspired by introns from cyanobacteria *Nostoc punctiforme*.^[^ ^36^ ^]^ Inteins have the capability to excise themselves from specific sequences and join the exteins to form the final proteins. We divided NiCas12b into two parts, namely N‐NiCas12b and C‐NiCas12b. Three split sites were chosen: 540Ser and 541Asn (540N+541C), 631Lys and 632Ala (631N+632C), or 756Lys and 757Thr (756N+757C) (Figure 4e). The N‐intein was fused to N‐NiCas12b, and the C‐intein was fused to C‐NiCas12b.

Following transfection of Hepa1‐6 cells with the resulting N‐NiCas12b and C‐NiCas12b, along with sgRNA1 expressing plasmids, genomic DNA was isolated for targeted deep sequencing. Results indicated that 540N+541C and 756N+757C displayed similar activity, surpassing that of 631N+632C (Figure 4F). Notably, all split‐NiCas12b variants exhibited lower activity compared to full‐length NiCas12b (Figure 4f). Genome editing with 756N+757C, despite the reduced activity, effectively lowered *Pcsk9* expression at both mRNA and protein levels (Figure 4g,h). Consequently, 756N+757C was selected for subsequent studies based on these findings.

### 2.8 In Vivo Genome Editing of NiCas12b Targeting *Pcsk9* in Mouse Liver

**Figure 5:**
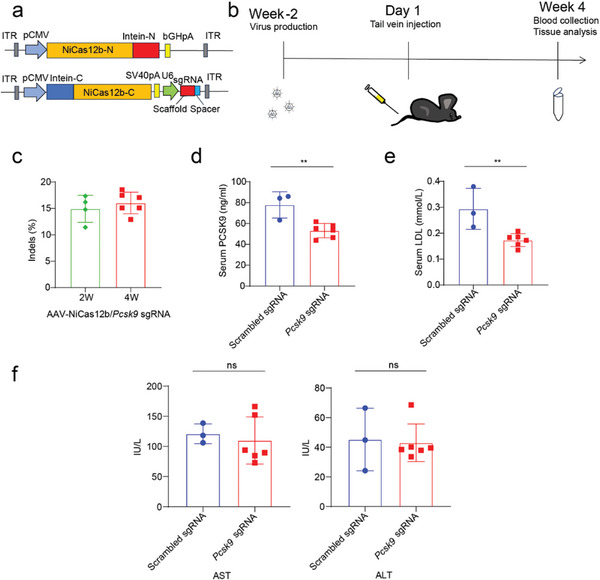
AAV8‐delivery of NiCas12b for in vivo genome editing. a) Schematic diagram of the AAV8‐NiCas12b vectors. CMV, cytomegalovirus. U6, U6 promoter. ITR, inverted terminal repeats. b) The procedure of in vivo genome editing in mice. Viruses are administered intravenously via tail vein injection in 3 weeks C57BL/6J mice. c) Indel efficiencies are measured at 2 and 4 weeks after genome editing using AAV‐NiCas12b in mice. Each dot represents a mouse. d) Serum PCSK9 protein levels are determined by ELISA after 4 weeks genome editing using AAV‐NiCas12b. The scrambled sgRNA group was used as a control. Each dot represents a mouse. e) Serum LDL cholesterol levels are determined by ELISA after 4 weeks genome editing using AAV‐NiCas12b. The scrambled sgRNA group was used as a control. Each dot represents a mouse. f) Liver function tests, including aspartate aminotransferase (AST) and alanine aminotransferase (ALT) in the AAV‐NiCas12b *Pcsk9* sgRNA group and scrambled sgRNA group. Each dot represents a mouse.

To evaluate the genome‐editing efficacy of the split‐NiCas12b system in vivo, we introduced split‐NiCas12b‐N into one AAV8 vector and split‐NiCas12b‐C with its sgRNA into another AAV8 vector (**Figure** **5**a). We administered these vectors to 3‐week‐old male C57BL/6 mice via intravenous injection through the lateral tail vein. Each mouse received 1 × 10^14^ GC kg^−1^ of AAV vector genomes in a total injection volume of 100 µL. Mice injected with scrambled sgRNA were used as a control. Liver tissues were collected at 2‐ and 4‐week intervals post injection, and genomic DNA was extracted for targeted deep sequencing (Figure 5b). At both time points, we observed indels at the target locus, accounting for 14.9% and 16.0%, respectively (Figure 5c). After 4 weeks, to track the serum levels of PCSK9 and total cholesterol, mice were fasted overnight for 12 h. Blood samples were collected, and enzyme linked immunosorbent assay (ELISA) was employed to determine serum PCSK9 dosage and LDL levels. We recorded a 31.7% reduction in serum PCSK9 (Figure 5d) and a 41.0% decrease in LDL levels (Figure 5e). These findings underscore the potential of NiCas12b for in vivo genome editing.

In a therapeutic context, targeting specificity is important for safety. We tried to find off‐target sites by GUIDE‐seq in Hepa1‐6 cells and then tested these sites in the liver by targeted deep sequencing. However, GUIDE‐seq did not detect off‐target sites for sgRNA1 (Figure S14, Supporting Information). The histological examination of the liver by hematoxylin and eosin (H&E) staining showed no signs of inflammation (Figure S15a,b, Supporting Information). Mice treated with AAV revealed normal liver function compared to untreated mice, reflected by normal transaminase levels, including aspartate aminotransferase (AST) and alanine aminotransferase (ALT) (Figure 5f).

To comprehensively evaluate off‐target effects on a genome‐wide scale, we performed whole‐genome sequencing (WGS) of liver DNA samples from two mice after 4 weeks of NiCas12b editing. Mice injected with scrambled sgRNA served as controls (Figure S16, Supporting Information). We detected 5 single nucleotide variants (SNVs) and 11 indels in edited mouse 1 and 11 SNVs and 21 indels in edited mouse 2 (Tables S4–S7, Supporting Information).

To determine whether these SNVs and indels were induced by NiCas12b, we used Cas‐OFFinder^[^ ^37^ ^]^ to search for potential off‐target sites with up to five mismatches and identified four potential off‐target sites (Table S8, Supporting Information). None of the mutations occurred at these potential off‐target sites (including the 500‐bp upstream and downstream regions). These results suggest that the NiCas12b system operates safely for in vivo genome editing.

## 3 Conclusion

CRISPR‐Cas12b represents the third family of CRISPR‐Cas systems employed for genome editing, yet only a limited number of Cas12b nucleases have been developed for this purpose.^[^ ^23^, ^38^ ^]^ As more organism genomes are sequenced, novel CRISPR‐Cas12b systems continue to be identified. In this study, we identified four Cas12b nucleases that exhibit activity in mammalian cells, thereby expanding the Cas12b toolbox. Notably, DtCas12b and NiCas12b demonstrated significantly higher activity than AaCas12b, enabling efficient genome editing across various cell types. Additionally, DtCas12b and NiCas12b exhibited distinct target sequence preferences, providing opportunities for site‐specific genome editing.

In this study, we screened 15 Cas12b orthologs and found that four exhibited activity in mammalian cells. Recently, we screened 82 Cas9 nucleases, with five showing activity in mammalian cells.^[^ ^39^ ^]^ Previously, Zetsche et al. screened 16 Cas12a nucleases and discovered that two were functional in mammalian cells.^[^ ^5^ ^]^ These data demonstrate that only a small fraction of bacterial nucleases can function efficiently in mammalian cells. One possible reason for this is the significant differences in post‐transcriptional modifications of proteins and RNA between mammalian cells and bacteria. Such modifications include glycosylation, phosphorylation, acetylation, ubiquitination, and methylation of specific bases.^[^ ^40^, ^41^ ^]^ These modifications may influence the structure of Cas nucleases and sgRNAs, thereby affecting their function.

While CRISPR‐Cas12b has been applied for genome editing in a range of plants^[^ ^42^, ^43^ ^]^ and human cell lines,^[^ ^23^, ^38^ ^]^ its therapeutic potential in vivo has not been thoroughly explored. In this study, we demonstrate that the split‐NiCas12b can be delivered via AAV for in vivo genome editing in mice. NiCas12b achieved an indel rate of 16.0% in editing the *Pcsk9* gene, comparable to several previous studies.^[^ ^44^, ^45^ ^]^ Subsequently, a reduction in serum PCSK9 and LDL levels was observed. Importantly, no off‐target cleavage was detected, and there were no adverse effects on liver functions post genome editing. These findings underscore the safety and specificity of NiCas12b for in vivo genome editing applications.

The long‐term safety is especially important for gene therapy of diseases, which frequently require life‐long efficacy and safety, especially in viral vector‐mediated in vivo gene therapy. Although we analyzed the off‐target effect via WGS after 1 month editing in mice, some experiments still need to do in the future before clinical translation. The important part of future study is the long‐term experiments with treated mice with a follow‐up of up to 6 months. Not only the editing efficacy but the off‐target effect and inflammatory response need to be test. During 6 month NiCas12b editing, the serum samples will be collected for testing the PCSK9 protein and LDL cholesterol levels by ELISA at least 1 month a time. The DNA samples of livers will be collected and genomic DNA was extracted for editing efficacy and off‐target effect tests. Also, the inflammatory response can be confirmed by histological examination of the 6 months edited livers after H&E staining.

Cas protein size is a major concern when using AAV (packaging size <4.7 kb) for gene therapy. To address this issue, one strategy is to identify small Cas nucleases, such as SaCas9,^[^ ^2^ ^]^ SauriCas9,^[^ ^6^ ^]^ Nme2Cas9,^[^ ^15^ ^]^ Cas12j‐8,^[^ ^16^ ^]^ and Cas12f.^[^ ^46^ ^]^ Another strategy is to split Cas proteins into two parts and deliver them using two AAV vectors. For instance, SpCas9 and base editors have been successfully delivered by two AAV vectors for in vivo genome editing.^[^ ^47^, ^48^ ^]^ NiCas12b has a large genome size, which limits its delivery by a single AAV vector. In this study, we demonstrate that NiCas12b can be delivered by two AAV vectors for in vivo genome editing. Additionally, the size of NiCas12b is not a limitation when delivered by non‐AAV methods, such as microinjection, electroporation, polymers, liposomes, lipid nanoparticles (LNP), gold particles, inorganic nanoparticles, exosomes, cell‐penetrating peptides, adenovirus, lentivirus, and retrovirus.^[^ ^49^, ^50^ ^]^ Since the modified LNP could help to deliver Cas9/sgRNA ribonucleoprotein complexes (RNPs) into cells and edit tissues including muscle, brain, liver, and lungs,^[^ ^51^ ^]^ the NiCas12b may edit the multiple targets by the similar strategies using LNP in vivo. The key problem of this delivery method is modifying the established dilution methods for higher packing efficiency and delivery capability. The editing efficacy and off‐target effect tests also need to perform. These alternative delivery methods will facilitate the application of NiCas12b for in vivo genome editing.

## 4 Experimental Section

### Plasmid Construction

The fragments of Cas12b candidates were synthesized by HuaGene (Shanghai, China) and inserted into the pAAV backbone. Human codon optimization was performed for the Cas12b ortholog fragments. The tracrRNA:crRNAs were synthesized by HuaGene and cloned into the pUC19 backbone. The pAAV‐NiCas12b‐541C‐U6, pAAV‐NiCas12b‐632C‐U6, pAAV‐NiCas12b‐757C‐U6, pAAV‐NiCas12b‐540N, pAAV‐NiCas12b‐631N, and pAAV‐NiCas12b‐756N plasmids were constructed using the ClonExpress Ultra One Step Cloning Kit (Vazyme). The primers are listed in Table S1 (Supporting Information). The target sites used are listed in Table S2 (Supporting Information). The sequences used in this study are listed in Table S3 (Supporting Information).

### Cell Culture and Transfection

Dulbecco's modified Eagle's medium (DMEM) supplemented with 10% fetal bovine serum (FBS, Gibco) and streptomycin (100 U mL^−1^) was used to culture HEK293T, AC16, Hepa1‐6, Neuro‐2a (N2a), and HeLa cells at 37 °C and 5% CO_2_. HCT116 cells were maintained in McCoy's 5A medium (Gibco) supplemented with 10% FBS. GFP reporter cells were plated in 10 cm dishes and transfected with Cas12b (12 µg) and crRNA plasmid (6 µg) using Lipofectamine 2000 (Life Technologies) when reaching ≈70% confluence for PAM screening. To test the editing capability of Cas12b at endogenous sites, cells were seeded in 48‐well plates and transfected with Cas12b plasmid (300 ng) and crRNA plasmid (200 ng) using Lipofectamine 2000 (Life Technologies). To test specificity, GFP reporter cells were cultured in 48‐well plates and transfected with Cas12 plasmid (300 ng) and crRNA plasmid (200 ng) using Lipofectamine 2000 (Life Technologies).

### Analysis of PAM Sequence in the GFP Activation Assay

The methods of the construction of the GFP activation system were previously reported.^[^ ^16^ ^]^ The Cas12b specificity was tested using the GFP activation assay. Plasmids of Cas12b candidates and crRNA were transfected into the GFP reporter cell library using Lipofectamine 2000 (Life Technologies). Following genome editing at the target site, random insertions or deletions (Indels) occurred, resulting in in‐frame mutations in the GFP sequence. This led to the activation of GFP expression in a subset of cells, resulting in GFP‐positive cells. Flow cytometry was employed to sort out the GFP‐positive cells, and their genomes were subsequently extracted for further analysis, such as deep sequencing. For PAM analysis, target sequences containing in‐frame mutations were utilized. The 5‐bp random sequence was extracted and visualized using WebLog3 and PAM wheel charts to display PAMs. The relative primers used in this analysis can be found in Table S1 (Supporting Information).

### Genome Editing at Endogenous Sites

HEK293T cells were cultured in a 48‐well plate and transfected with the Cas12b plasmid (300 ng) + crRNA plasmids (200 ng) using Lipofectamine 2000 (Life Technologies). Cells were selected with puromycin and collected 5–7 days after transfection. The transfection was performed with Cas12b plasmid (600 ng) + crRNA plasmids (400 ng) using Lipofectamine 3000 (Life Technologies). Cells were selected with puromycin and collected 7 days after transfection. Genomic DNA was isolated, and the target sites were PCR‐amplified by nested PCR and purified with the Gel Extraction Kit (Tiangen, DP219) for deep sequencing. After demultiplexing, the samples were analyzed using CRISPResso2.

### Test of Cas12b Specificity with the GFP Activation Assay

To test the specificity of Cas12b, a GFP reporter cell line with a 5′‐GTTTT‐3′ PAM was generated. HEK293T cells were seeded into 48‐well plates and transfected with 300 ng of Cas12b plasmids and 200 ng of sgRNA plasmids using Lipofectamine 2000. Five days after transfection, the GFP‐positive cells were harvested by digestion and centrifugation at 800 rpm for 4 min. The cells were resuspended in phosphate‐buffered saline (PBS). The cells were then resuspended in PBS. Finally, the cells were analyzed using a Calibur instrument (BD), and the obtained data were analyzed using FlowJo software. This analysis allowed for the assessment of the specificity of Cas12b by measuring the GFP expression in the transfected cells.

### GUIDE‐seq

In this study, a GUIDE‐seq experiment was conducted with certain modifications to the original protocol, as described in reference.^[^ ^27^ ^]^ On the day of the experiment, 2 × 10^5^ HEK293T cells per target site were harvested and washed with PBS. Subsequently, they were transfected with 500 ng of Cas12b plasmid, 500 ng of sgRNA plasmid, and 100 pmol of annealed GUIDE‐seq oligonucleotides using the Neon Transfection System. For HEK293T cells, the electroporation parameters were set as follows: voltage, 1150 V; pulse width, 20 ms; and the number of pulses, 2. For Hepa1‐6 cells, the electroporation parameters were as follows: voltage, 1300 V; pulse width, 20 ms; and number of pulses, 1. Genomic DNA was extracted with the DNA Extraction Kit (Tiangen, DP304) 6 days after electroporation according to the manufacturer's protocol. Then, genome fragmentation, adapter ligation, and library construction were performed. The samples were subjected to the PCR amplification step. Samples were sent to Mingma Technologies (China, Shanghai) for deep sequencing. After demultiplexing, the samples were analyzed.

### RT‐qPCR

Total RNAs were extracted using RNA isolater Total RNA Extraction Reagent (Vazyme, R401‐01), and RT was performed using HiScript Q RT SuperMix for qPCR (Vazyme, R123‐01). qPCR was performed to measure the expression of *Mouse Pcsk9* mRNA mRNA using Taq Pro Universal SYBR qPCR Master Mix (Vazyme, Q712‐02). Primers used for qPCR are listed in Table S1 (Supporting Information).

### Western Blot

Cells were washed with cold PBS and then lysed in cold lysis buffer consisting of 150 mm NaCl, 50 mm Tris‐Cl (pH 7.4), one mm ethylenediaminetetraacetic acid (EDTA), 1% Triton, and complete mini tablet‐protease inhibitor cocktail tablets. The lysates were subjected to protein separation using 10% sodium dodecyl sulfate polyacrylamide gel electrophores (SDS‐PAGE) and subsequently transferred to polyvinylidene fluoride (PVDF) membranes. Following the transfer, the membranes were blocked with 5% defatted milk in tris buffered saline (TBS) with Tween‐20 (TBST) and then incubated overnight at 4 °C with one of the following primary antibodies: anti‐GADPH (Yeason, 30201ES60), anti‐FLAG (Yeason, 30503ES80), and anti‐PCSK9 (Abcam, ab185194). The next day, the membranes were washed three times with TBST and incubated with the corresponding antimouse (Proteintech, SA00001‐1) or antirabbit (Proteintech, SA00001‐2) secondary antibodies at a 1:10 000 dilution at room temperature for 1 h. Signal intensity was quantified using ImageJ software.

### AAV Vector Construction

The sgRNA1 target to murine Pcsk9 exon 1 was selected. The sgRNA (GTACCGGGGCCCGTTAATGTTTAA) was cloned into the pAAV[Exp] plasmid (from VectorBuilder, China). The pAAV‐NiCas12b‐N vector was constructed by infusion cloning and includes a cytomegalovirus (CMV) promoter, NiCas12b‐N, and intein‐N. Similarly, the pAAV‐NiCas12b‐C‐sgRNA1 and pAAV‐NiCas12b‐C‐non‐target vectors were constructed via infusion cloning. The pAAV‐NiCas12b‐C‐sgRNA1 vector contains a CMV promoter, NiCas12b‐C, intein‐C, and a U6‐Pcsk9 sgRNA1 targeting a specific region in exon 1. The pAAV‐NiCas12b‐C‐non‐target vector includes a CMV promoter, NiCas12b‐C, intein‐C, and a scrambled nontarget sgRNA, which does not target any region in the murine genome, serving as a control group.

### AAV Production and Purification

Following the preparation of the pHelper, AAV8 Rep‐Cap plasmid, and pAAV[Exp] (pAAV‐NiCas12b‐N, pAAV‐NiCas12b‐C‐sgRNA1, and pAAV‐NiCas12b‐C‐non‐target), HEK293T cells were used for AAV packaging. HEK293T cells, grown to 80–90% confluence in DMEM supplemented with 10% FBS, were triple transfected with pHelper, pAAV Rep‐Cap plasmids, and pAAV[Exp] using Lipofectamine 2000 in 10 cm dishes. Before transfection, the medium was replaced with Opti‐MEM. Six hours post transfection, the medium was changed back to DMEM with 10% FBS. Two days post transfection, the media and cells were collected and centrifuged at 2000 × *g* at 4 °C for 20 min. The AAV‐containing supernatant was carefully transferred to a clean tube and filtered through a 0.45 µm PVDF membrane (Millex‐HV, Millipore). Cells were lysed in 180 µL of AAV Lysis buffer (an acidic buffer), followed by three cycles of freezing (in liquid nitrogen) and thawing (in a 37 °C water bath) to release AAVs from the cells. The AAV‐containing supernatant was concentrated using PEG8000, and the PEG‐precipitated AAVs were collected by centrifugation at 2000 × *g* at 4 °C for 30 min. The AAV stock solution was treated with 50 U mL^−1^ Benzonase at 37 °C for 45 min. After a 20 mins centrifugation, the supernatant was collected. For in vivo use, AAVs were further purified. The 2 mL of 1.5 g mL^−1^ CsCl was added to a clean tube, followed by the slow addition of 3 mL of 1.35 g mL^−1^ CsCl. After adding 5 mL of the AAV suspension, the mixture was centrifuged at 4000 × *g* at 4 °C for 2 h. The white AAV band, up to 1.5 g mL^−1^ CsCl, was collected into an ultrafiltration dialysis tube and centrifuged at 4000 × *g* at 4 °C for 6 min. This process was repeated twice, and the purified AAV solution was transferred to a clean tube. The titer of the AAV was determined by RT‐qPCR using the AAVpro Titration Kit Ver.2 (Takara).

### Animal Injection and Processing

All animal procedures were conducted in accordance with the guidelines and approval of the Ethics Committee of the School of Life Sciences, Fudan University. Humane care was provided to all animals throughout the study. The AAV vector was intravenously delivered to 3‐week‐old male C57BL/6J mice via lateral tail vein injection, with dosages adjusted to 100 µL (2 × 10^14^ GC kg^−1^ per mouse) using sterile PBS, pH 7.4. Animals were not subjected to immunosuppression or any special treatment before or during the experiment, except for fasting prior to submandibular vein bleeds.

To track the serum levels of Pcsk9 and total cholesterol, animals were fasted overnight for 12 h prior to blood collection by submandibular vein bleeds (no more than 100 µL or 10% of total blood volume per week). After the blood was allowed to clot at room temperature, the serum was separated by centrifugation at 1000 × *g* for 15 min and stored at −80 °C for later analysis. The median lobe of the liver was excised and fixed in 4% paraformaldehyde fix solution (4% PFA) for histological analysis. The remaining lobes were sliced into small blocks and frozen for subsequent DNA extraction.

### Histology and Serum Analysis

Tissues were fixed using 4% PFA at 4 °C overnight and dehydrated the next day before paraffinization. Paraffin blocks were cut into 5 µm thick sections, deparaffinized with xylene, and rehydrated. Sections were stained for H&E and examined for histopathological changes.

Serum levels of Pcsk9 were determined using the Mouse PCSK9 ELISA Kit (Elabscience, E‐EL‐M0634c) following the manufacturer's instructions. Similarly, the Mouse LDL ELISA Kit (Elabscience, E‐EL‐M1363c) was utilized to measure LDL levels in the serum. The measurements of serum ALT and AST were carried out using the Alanine Aminotransferase Activity Assay Kit (Elabscience, E‐BC‐K235‐M) and the Aspartate Aminotransferase Activity Assay Kit (Elabscience, E‐BC‐K236‐M), respectively.

### DNA Isolation from Tissue and Indel Analysis

Genomic DNA from mouse liver tissues was mechanically ground using TGrinder (Tiangen, 154119‐001) and subsequently isolated using the DNA Extraction Kit (Tiangen, DP304) according to the manufacturer's protocol. Target sites were amplified by PCR using KOD FX (TOYOBO, KFX‐101) for 25 cycles. Then, it was amplified with primers containing sequencing adaptors for another 15 cycles. The products were gel purified. Samples were sent to Mingma Technologies (China, Shanghai) for deep sequencing. After demultiplexing, the samples were analyzed using CRISPResso2.

### Quantification and Statistical Analysis

GraphPad Prism was used for statistical analyses and graph generation. All experiments were repeated at least three times. For comparisons between two groups, the *t*‐test assuming a two‐tailed distribution was employed. Data were expressed as the mean ± standard deviation (SD) with dots to show the actual data points. Significance was defined as #*p* > 0.05, *p < 0.05, **p < 0.01, ***p < 0.001, and ****p < 0.0001.

## Conflict of Interest

Authors are applying for a patent related to the work.

## Author Contributions

Y.Z. performed experiments and wrote the manuscript. J.W. provided experimental guidance. H.W. revised the manuscript. Y.W. wrote and revised the manuscript.

## Supporting information

Supporting Information

Supplemental Table 1

Supplemental Table 2

Supplemental Table 3

Supplemental Table 4

Supplemental Table 5

Supplemental Table 6

Supplemental Table 7

Supplemental Table 8

## Data Availability

The data that support the findings of this study are available in the supplementary material of this article.
