# Supplementary material for: Characterization of NiCas12b for In Vivo Genome Editing
Source: Adv Sci (Weinh). 2024 Jul 30;11(36):2400469. doi: 10.1002/advs.202400469 (PMC11423069; doi:10.1002/advs.202400469)
Supplement: Supplementary file 1 — Supporting Information [file ADVS-11-2400469-s003.docx]

Supporting Information

**Characterization of NiCas12b for *in vivo* Genome Editing**

*Yunqian Zhang, Jingjing Wei,* *Hongyan Wang*, and Yongming Wang**

Yunqian Zhang

Shanghai Key Laboratory of Metabolic Remodeling and Health, Institute of Metabolism and Integrative Biology, State Key Laboratory of Genetic Engineering at School of Life Sciences, Fudan University, Shanghai 200438, China

Jingjing Wei

Center for Medical Research and Innovation, Shanghai Pudong Hospital, Fudan University Pudong Medical Center, Fudan University, Shanghai 200438, China

Hongyan Wang

Obstetrics & Gynecology Hospital, State Key Laboratory of Genetic Engineering, Shanghai Key Laboratory of Metabolic Remodeling and Health, Institute of Metabolism and Integrative Biology, Fudan University, Shanghai 200438, China

Children's Hospital, Fudan University, Shanghai 201102, China

E-mail: [wanghy@fudan.edu.cn](mailto:wanghy@fudan.edu.cn)

Yongming Wang

Center for Medical Research and Innovation, Shanghai Pudong Hospital, Fudan University Pudong Medical Center, Fudan University, Shanghai 200438, China

Shanghai Engineering Research Center of Industrial Microorganisms, Shanghai, 200438, China.

E-mail: [ymw@fudan.edu.cn](mailto:ymw@fudan.edu.cn)

Keywords: CRISPR-Cas, NiCas12b, PCSK9

**Figure S1.** Phylogenetic tree of AaCas12b orthologs. Three previously characterized Cas12b orthologs are marked in blue.

**Figure S2.** Schematic illustration of the genomic architecture of CRISPR-Cas12b. The AaCas12b, Cas1, Cas2, tracrRNA, and CRISPR arrays are indicated.

**Figure S3.** Alignment of repeat and tracrRNA sequences by the Vector NTI. a) Sequence alignment of repeat sequences among Cas12b orthologs. b) Sequence alignment of tracrRNA sequences among Cas12b orthologs.

**Figure S4.** Analysis of secondary RNA structures for Cas12b scaffolds. These structures are generated using the RNA Folding Form V2.3c (http://www.unafold.org/mfold/applications/rna-folding-form-v2.php). The red boxes indicate the nucleotides that pair with the 3’ end of the repeat.

**Figure S4 continued.**

**Figure S5.** Test of the Cas12b spacer length. a) The schematic diagram illustrates the sgRNA expression construct, with a panel of spacers of varying lengths shown below. b) The editing efficiencies of four Cas12b orthologs (n=3).

**Figure S6.** Engineer of the AlicCas12b scaffold. a) Secondary structures of wild-type (WT) and engineered AlicCas12b scaffolds (V1). The red boxes indicate the nucleotides that pair with the 3’ end of the repeat. The red arrow indicates the modification site. b) Indel efficiencies of each scaffold variant (n=3).

**Figure S7.** Engineer of the DtCas12b scaffold. a) Secondary structures of WT and engineered DtCas12b scaffolds (V1-V2). The red boxes indicate the nucleotides that pair with the 3’ end of the repeat. The red arrows indicate the modification site. b) Indel efficiencies of each scaffold variant (n=3).

**Figure S8.** Engineer of the NiCas12b scaffold. a) Secondary structures of WT and engineered NiCas12b sgRNAs (V1-V4). The red boxes indicate the nucleotides that pair with the 3’ end of the repeat. The red arrows indicate the modification site. b) Indel efficiencies of each scaffold variant (n=3).

**Figure S9.** Engineer of the ThCas12b scaffold. a) Secondary structures of wild-type (WT) and engineered ThCas12b scaffolds (V1). The red boxes indicate the nucleotides that pair with the 3’ end of the repeat. The red arrow indicates the modification site. b) Indel efficiencies of each scaffold variant (n=3).

**Figure S10.** Analysis of Cas12b activity in the mouse N2a cells. a) The editing efficiency of AaCas12b, DtCas12b, NiCas12b, and AlicCas12b in mouse N2a cells is measured by targeted deep sequencing (n = 3). The sequences of target sites are shown on the left. The PAM are marked in red. AaCas12b, DtCas12b, and NiCas12b use 24-nt spacers. AlicCas12b uses 21-nt spacer. b) Quantification of editing efficiencies in N2a cells. For post hoc analysis, repeated-measures (RM) one-way analysis of variance (ANOVA) test followed by Fisher’s least significant difference test is used.

**Figure S11.** NiCas12b enables genome editing in different cell lines. a) NiCas12b enables genome editing in HeLa cells. b) NiCas12b enables genome editing in AC16 cells. c) NiCas12b enables genome editing in Hct116 cells. The PAM are marked in red.

**Figure S12.** The procedure of allele-specific genome editing. a) Schematic of the lentivirus vector containing a DNA fragment carrying disease-causing mutations. b) The lentivirus stably integrated into the chromosome in HEK293T cells, followed by FACS sorting. Both NiCas12b and SpCas9 were used to edit the integrated targets. Targeted deep sequencing was used to analyze indel rates for endogenous WT allele and mutant allele (integrated targets).

**Figure S13.** Allele-specific editing of dominant-negative mutations by NiCas12b and SpCas9. (a-f) Allele-specific genome editing was performed at the following loci: *F11* c. 1207G>A (a), *F11* c. 127G>A (b), *KCNC3* c. 1268G>A (c), *MYH7* c. 767G>A (d), *TP53* c. 733G>A (e), and *TP53* c. 742C>T (f). The sequences of the mutant allele and sgRNAs are shown on the left. NiCas12b PAMs are shown in red; NiCas12b spacers are indicated by blue arrows. SpCas9 PAMs are shown in blue; SpCas9 spacers are indicated by red arrows. The mutations are indicated by small green letters.

**Figure S14.** Off-target effects of NiCas12b and intein-mediated split NiCas12b (756N+757C) are analyzed by GUIDE-seq. The Pcsk9-targeting sgRNA-1 is used. Read numbers for on- and off-targets are shown on the right. Mismatches compared with the on-target site are shown.

**Figure S15.** Histological analysis of the liver is performed with H&E staining at four weeks after injection. (a) Histological analysis of the liver for three wild-type mice. (b) Histological analysis of the liver for six AAV-NiCas12b treated mice. Scale bar, 250 μm.

**Figure S16.** Schematic diagram of off-target analysis by whole-genome sequencing. After four weeks of NiCas12b editing, we extracted mouse liver genomic DNA and then break into 350-450bp fragments. After end repair and adaptor ligation, the fragments were amplified by PCR. The MGI DNBSEQ-T7 platform were used to paired-end sequencing after library quality control. We first normalized the WGS data by deleting regions of low-quality reads using FastQC software (https://www.bioinformatics.babraham.ac.uk/projects/fastqc/). The sequencing depth was approximately х43–х51. The high-quality reads were aligned to the reference mouse genome GRCm38 (mm10) using BWA (v0.7.17). After data processing, false positives were removed, and candidate mutations for each sample were acquired. The Sentieon (202010.01) was used to detect the SNVs and Indels and Annovar was used for annotation. To determine whether these SNVs and indels were induced by NiCas12b, we used Cas-OFFinder to search for potential off-target sites with up to five mismatches.
